# Neuropeptide Y1 receptor antagonist promotes osteoporosis and microdamage repair and enhances osteogenic differentiation of bone marrow stem cells via cAMP/PKA/CREB pathway

**DOI:** 10.18632/aging.103129

**Published:** 2020-05-07

**Authors:** Weixin Xie, Fan Li, Yi Han, Yi Qin, Yuan Wang, Xiaoying Chi, Jie Xiao, Zhanchun Li

**Affiliations:** 1 Department of Anesthesiology, Renji Hospital, School of Medicine, Shanghai Jiaotong University, Shanghai 200127, China; 2 Department of Orthopaedic Surgery, Renji Hospital, School of Medicine, Shanghai Jiaotong University, Shanghai 200127, China

**Keywords:** osteoporosis, neuropeptide Y, bone microstructure, bone mcirodamage, bone marrow stromal cells

## Abstract

Osteoporosis is a common metabolic bone disorder in the elderly population. The accumulation of bone microdamage is a critical factor of osteoporotic fracture. Neuropeptide Y (NPY) has been reported to regulated bone metabolism through Y1 receptor (Y1R). In this study the effects and mechanisms of Y1R antagonist on prevention for osteoporosis were characterized. In the clinical experiment, compared with osteoarthritis (OA), osteoporosis (OP) showed significant osteoporotic bone microstructure and accumulation of bone microdamage. NPY and Y1R immunoreactivity in bone were stronger in OP group, and were both correlated with bone volume fraction (BV/TV). In vivo experiment, Y1R antagonist significantly improved osteoporotic microstructure in the ovariectomized (OVX) rats. And Y1R antagonist promoted RUNX2, OPG and inhibit RANKL, MMP9 in bone marrow. In vitro cell culture experiment, NPY inhibited osteogenesis, elevated RANKL/OPG ratio and downregulated the expression of cAMP, p-PKAs and p-CREB in BMSCs, treated with Y1R antagonist or 8-Bromo-cAMP could inhibit the effects of NPY. Together, Y1R antagonist improved the bone microstructure and reduced bone microdamage in OVX rats. NPY-Y1R could inhibit osteoblast differentiation of BMSCs via cAMP/PKA/CREB pathway. Our findings highlight the regulation of NPY-Y1R in bone metabolism as a potential therapy strategy for the prevention of osteoporosis and osteoporotic fracture.

## INTRODUCTION

Osteoporosis is one of the most common metabolic bone disorders in the elderly population. Postmenopausal women with osteoporosis are at high risk for osteoporotic fracture, which result in pain, dysfunction, and even death. Due to the rapid growth in the incidence and economic burden of osteoporotic fracture, it is reasonable to focus on the prevention and treatment of postmenopausal osteoporosis (PMO). Osteoporosis is diagnosed clinically by the measurement of bone mineral density (BMD), while BMD alone is not sufficient to explain fracture incidence. Bone quality generally refers to the effects of skeletal factors that affect fracture but are not accounted for by bone mass or BMD [1]. Studies have revealed that there are several characteristics of bone which have been proposed as critical factors of bone quality, including bone microdamage [2–4].

Bone microdamage is generally defined as damage to matrix which can be detected by light microscopy [5]. Microdamage occurs when bone tissues are under physiological loading, which can be repaired with a dynamic balance between the generation and repair of microdamage in healthy bone tissues [6]. Some diseases like PMO, which distinctly affects the bone metabolism and biomechanics, are able to break the balance and contribute to the accumulation of bone microdamage and fractures. We previously reported that fatigue loading caused significant increased microcrack density in osteoporotic bone, which suggested that insufficient bone structure might result in the susceptibility to fatigue loading and the accumulation of microdamage [7]. The accumulation of microdamage has been implicated as an important factor which contributes to osteoporotic fracture [6]. Reducing the bone microdamage is important to the prevention of osteoporotic fracture. Bone microdamage is generally repaired by the bone remodeling process [8], which is involved in a coupled system of bone formation and resorption. Therefore, the balance between bone formation and resorption has been implicated in the regulation of bone microdamage repair.

Bone is plentifully innervated by peripheral nerve fibers within the bone marrow [9]. Neuropeptides such as neuropeptide Y (NPY) are secreted by the peripheral nervous system, and the NPY system has emerged as one of the major regulators of bone homeostasis [10]. Of the receptors of NPY, the Y1 receptor (Y1R) and Y2 receptor (Y2R) are crucial for the regulation of bone metabolism [11]. Y1R has been demonstrated to be expressed in osteoblastic cells, bone marrow stromal cells (BMSCs) and osteocytes, while Y2R was unable to be detected on bone cells [12–14]. Therefore, NPY might mainly mediate its actions via Y1R in bone tissues. Germline Y1R knockout mice resulted in a high bone mass phenotype with elevated osteoblast activity and bone formation [15]. Moreover, BMSCs isolated from Y1R deletion mice showed increased proliferation and mineralization [14]. It was suggested that the Y1R played a critical role on BMSCs in the regulation of bone metabolism, which might be involved in the improvement of osteoporosis and repair of microdamage, while the mechanisms remain unclear.

The cAMP/PKA signaling pathway in bone cells is vital to bone metabolism. Studies have demonstrated that the activation of cAMP/PKA/CREB signaling pathway in human BMSCs [16] and osteoblasts [17, 18] contributed to the osteogenic effects with the upregulated level of runt-related transcription factor 2 (Runx2). On the other hand, some studies found that the activation of cAMP/PKA pathway in mesenchymal stem cells [19] and osteoblasts [20] resulted in the decreased ratio of receptor activator of the NF-kB ligand to osteoprotegerin (RANKL/OPG), which inhibited the osteoclastogenesis. NPY has been shown to contribute to a reduction in the levels of intracellular cAMP through Y1R and suppress the osteoblast differentiation [13]. Besides, NPY treatment strongly suppressed cAMP/PKA pathway and phosphorylation of CREB through Y1R in C3H10T1/2 cells which derived from mouse mesenchymal stem cells [21]. It prompted us to investigate whether NPY could affect the cAMP/PKA pathway in BMSCs through Y1R, which might regulate osteoblast and osteoclast differentiation.

In this study, we hypothesized that NPY inhibited the cAMP/PKA/CREB pathway in BMSCs through Y1R, which suppressed the osteoblast differentiation and enhanced the osteoclast activity. Y1R antagonist treatment played an anti-osteoporotic effect and promoted microdamage repair in ovariectomized rats. We aimed to characterize the effects and mechanisms of Y1R antagonist on the treatment for osteoporosis and microdamage.

## RESULTS

### OP group showed deteriorated bone microstructure and more microdamage than OA

**Figure 1:**
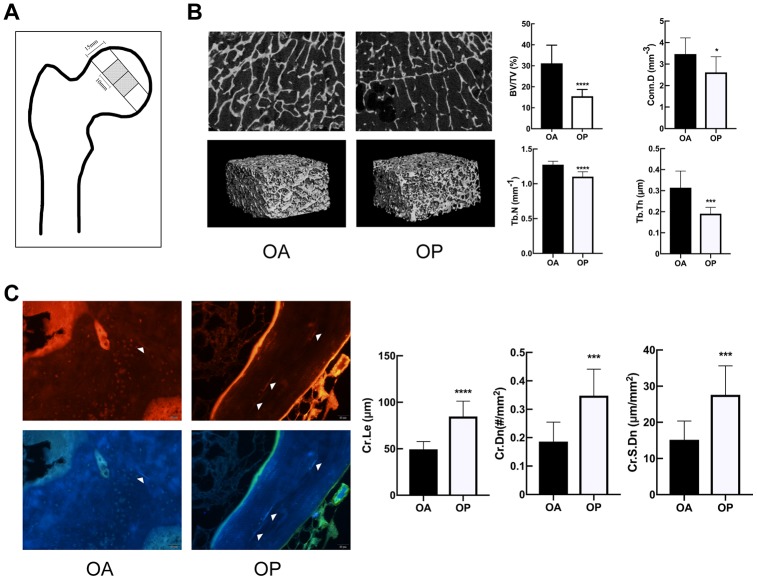
**OP group showed deteriorated bone microstructure and more microdamage than OA.** (**A**) The position of the samples for micro-CT and microdamage analysis within the femoral head. (**B**) Representative micro-CT images of tibias, and comparison of the microstructure parameters between two groups. (**C**) Fluorescent images and comparison of microdamage (white arrow head) in the OA group and OP group (x 200). The data are expressed as the means ± SD (n = 10 in each group). *P<0.05; ***P<0.001; ****P<0.0001 vs. SHAM by the unpaired two-tailed Student’s t-test.

There was no statistical difference with regard to the age of the patients between OA group and OP group (69.00±5.31 vs. 68.30±5.95 years, respectively, P=0.7845). The specimens were analyzed to perform the 3-D reconstruction of trabecular bone via micro-CT. As shown in Figure 1B, the OP group represented a marked deterioration in the microstructure of trabeculae. Compared to OA group, OP group showed significant decreases in BV/TV, Tb.Th, Tb. N and Conn.D but significant increase in Tb.Sp (Table 1).

**Table 1:** Comparison of the microstructural parameters of cancellous bone from OP and OA patients.

**Parameters**	**OA (n=10)**	**OP (n=10)**	**P value**
BV/TV (%)	31.200±8.664	15.500±3.224	0.000^#^
Tb.N (mm^-1^)	1.274±0.050	1.103±0.071	0.000^#^
Tb.Th (mm)	0.314±0.080	0.191±0.030	0.000^#^
Tb.Sp (mm)	0.721±0.061	0.841±0.076	0.001^#^
Conn.D (mm^-3^)	3.469±0.749	2.615±0.727	0.019^#^
SMI	0.912±0.426	1.761±0.306	0.000^#^

BV/TV, bone volume fraction; Tb.N, trabecular number; Tb.Th, trabecular thickness; Tb.Sp, trabecular spacing; Conn.D, connectivity density; SMI, structure model index. Data are expressed as mean ± SD (n = 10 in each group). ^#^P<0.05 vs. OA by the unpaired two-tailed Student’s t-test.

The microcracks of the specimens were imaged by red fluorescence and purple fluorescence. As shown in Figure 1C, significant increases were observed in the Cr.Le, Cr.Dn and Cr.S.Dn in OP group.

### NPY and Y1R were upregulated in subchondral cancellous bone of OP patients and OVX rats

**Figure 2:**
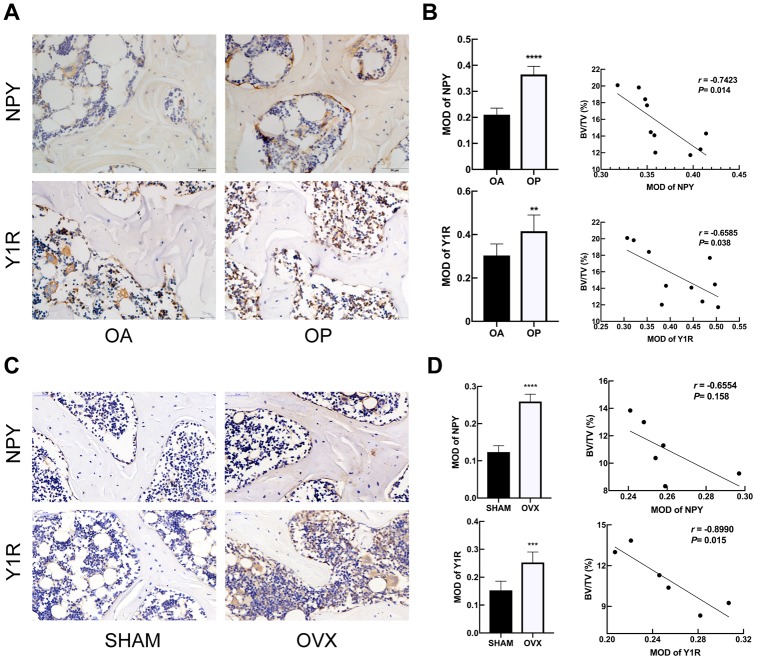
**NPY and Y1R were upregulated in subchondral cancellous bone of OP patients and OVX rats.** Immunohistochemical analysis of bone tissue between OA and OP group (**A**), and between SHAM and OVX group (**C**) for NPY and Y1R (x 400). Correlation between BV/TV and the expression of NPY and Y1R in OP group (**B**) and OVX group (**D**). The data are expressed as the means ± SD. r=correlation coefficient assessed by Pearson correlation analysis. **P<0.01; ***P<0.001; ****P<0.0001 by the unpaired two-tailed Student’s t-test.

The expression of NPY and Y1R in bone samples of patients and rats were evaluated by immunohistochemical analysis (Figure 2). Compared to OA group, the subchondral cancellous bone of OP patients exhibited stronger immunoreactivity for both NPY and Y1R (Figure 2A). Similarly, NPY and Y1R immunoreactivity in bone were stronger in the OVX rats than that in the SHAM rats (Figure 2C). In the OP patients and OVX rats, correlation analysis showed that the MOD of NPY and Y1R were negatively correlated with BV/TV, respectively (Figure 2B, 2D). It was suggested that the expressions of NPY and Y1R in bone tissue were associated with the bone metabolism in osteoporosis.

### Y1R antagonist treatment improved bone microstructure and decrease microdamage in OVX rats

**Figure 3:**
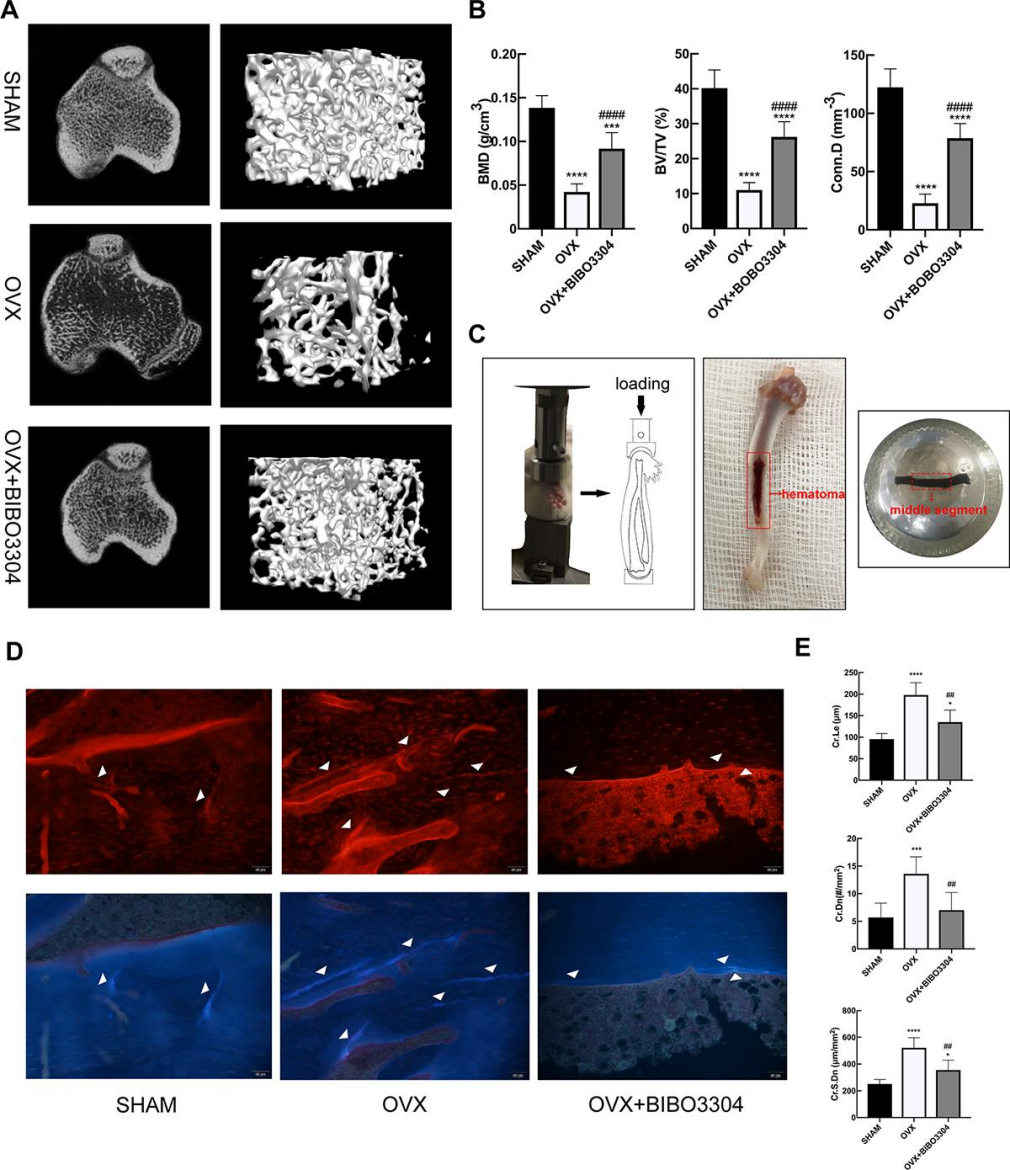
**Y1R antagonist treatment improved bone microstructure and decrease microdamage in OVX rats.** Representative micro-CT images of rat tibias (**A**) and quantitative analysis of bone microstructure (**B**). (**C**) In vivo fatigue loading model and the position of the sample for microdamage analysis in the rat tibia. Fluorescent images (**D**) and comparison (**E**) of microdamage (white arrow head) among groups (x 200). The data are expressed as the means ± SD (n = 6 in each group). *P<0.05; ***P<0.001; ****P<0.0001 vs. SHAM, and ^##^P<0.01; ^####^P<0.0001 vs. OVX by one-way ANOVA and Tukey’s post hoc test.

The micro-CT images of right tibias of rats clearly displayed the differences of microstructure among the groups (Figure 3A). As expected, the OVX rats exhibited remarkable reductions of BMD, BV/TV, Tb.Th, Tb.N and Conn.D. And the osteoporotic changes of bone microstructure in OVX rats were greatly improved after Y1R antagonist BIBO3304 treatment (Figure 3B) (Table 2).

**Table 2:** Comparison of the microstructural parameters of tibia from groups of rats.

**Parameters**	**SHAM**	**OVX**	**OVX+BIBO3304**
BV/TV (%)	40.200±5.182	11.010±2.133^*^	26.24±4.311*^#^
Tb.N (mm^-1^)	3.592±0.488	1.102±0.303^*^	2.700±0.489*^#^
Tb.Th (mm)	0.099±0.010	0.068±0.006^*^	0.093±0.008^#^
Tb.Sp (mm)	0.189±0.041	0.653±0.123^*^	0.293±0.093^#^
Conn.D (mm^-3^)	122.300±15.89	22.790±7.899^*^	78.530±12.680^*#^
SMI	1.331±0.373	2.515±0.161^*^	1.915±0.285^*#^

BV/TV, bone volume fraction; Tb.N, trabecular number; Tb.Th, trabecular thickness; Tb.Sp, trabecular spacing; Conn.D, connectivity density; SMI, structure model index. Data are expressed as mean ± SD (n = 6 in each group). ^*^P<0.05 vs. SHAM, ^#^P<0.05 vs. OVX by one-way ANOVA and Tukey’s post hoc test.

After in vivo fatigue loading, the middle segment of tibia specimens was obtained to evaluate the bone microdamage (Figure 3C). Cyclic loading induced microdamage and leaded to a significant increase in Cr.Le, Cr.Dn and Cr.S.Dn in OVX rats compared to the SHAM rats. In group treated with BIBO3304, the Cr.Le, Cr.Dn and Cr.S.Dn in OVX+ BIBO3304 rats were significantly decreased compared to OVX rats, suggesting that Y1R antagonist treatment prevented the load-induced increase in bone microdamage (Figure 3D–3E).

### Y1R antagonist treatment promoted bone formation and inhibit bone resorption in OVX rats

**Figure 4:**
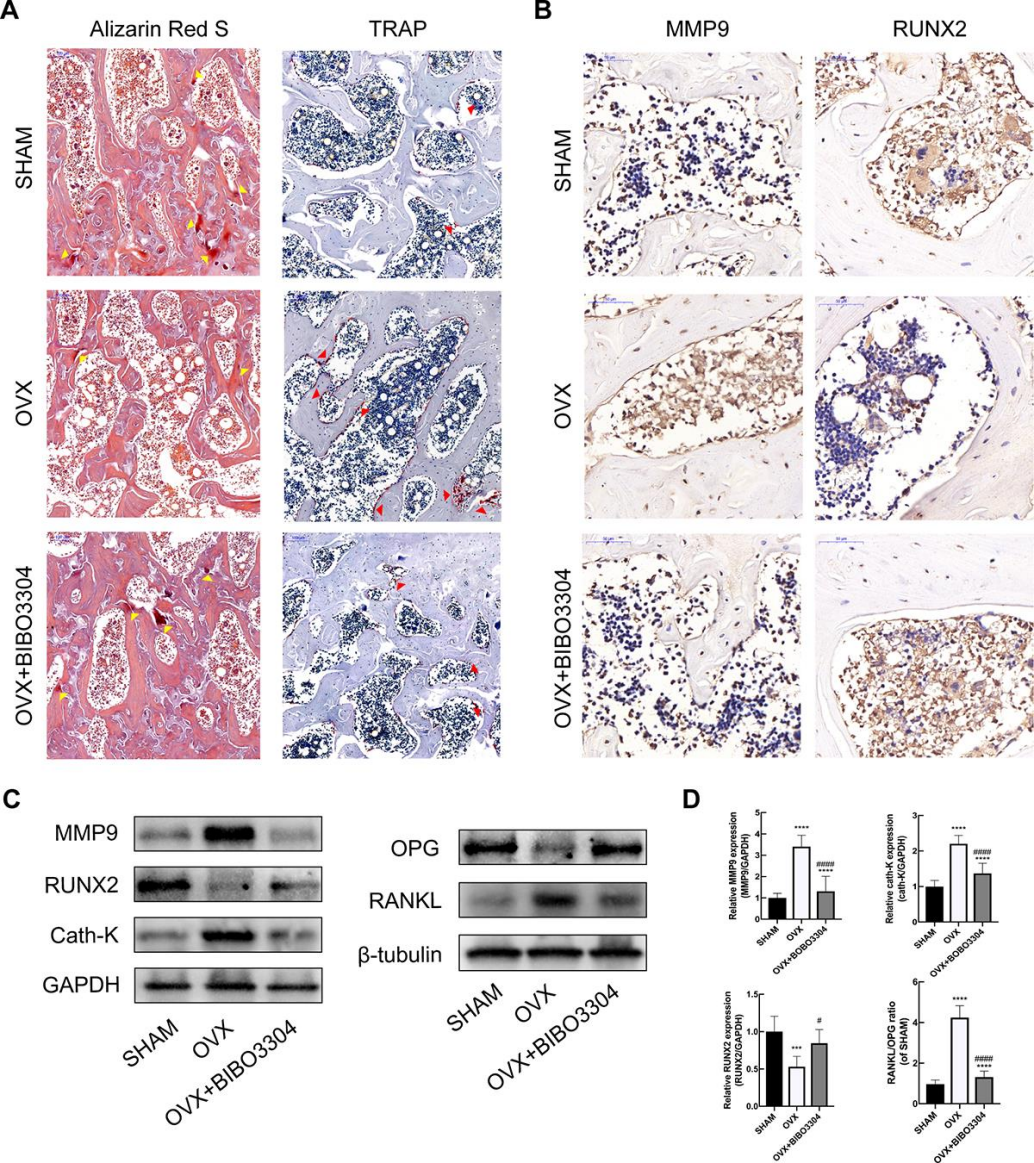
**Y1R antagonist treatment promoted bone formation and inhibit bone resorption in OVX rats.** (**A**) The Alizarin Red S (yellow arrow head) and TRAP staining (red arrow head) of bone tissues in groups. (**B**) Immunohistochemical analysis of bone tissue among groups for MMP9 and RUNX2 (x 400). (**C**, **D**) Western blotting results of MMP, RUNX2, Cath-K, OPG and RANKL expression in bone marrow from rat femurs. The data are expressed as the means ± SD (n = 6 in each group). ***P<0.001; ****P<0.0001 vs. SHAM, and ^#^P<0.05; ^####^P<0.0001 vs. OVX by one-way ANOVA and Tukey’s post hoc test.

The Alizarin Red S and TRAP staining were performed to evaluate the bone formation and resorption activities among groups. The calcified nodules in bone tissues would be stained by Alizarin Red S with the color of deep red, which reflects the activity of osteoblasts and bone formation [22]. As shown in Figure 4A, it is apparently that the area of calcified nodules was higher in BIBO3304 treatment groups than OVX group, which indicated that bone mineralization was improved with BIBO3304 treatment. TRAP as a marker enzyme of osteoclasts, was significantly down-regulated in the OVX rats with BIBO3304 treatment when compared with group OVX. The immunohistochemical analysis showed that OVX+BIBO3304 group exhibited stronger immunoreactivity for RUNX2 and lower for immunoreactivity for MMP9 than those in OVX group (Figure 4B).

The bone marrow from rat femurs was extracted and analyzed by western blotting. The results showed that the expression level of RUNX2 and OPG enhanced, and MMP9 and Cath-K decreased apparently in OVX+BIBO3304 group than those in the OVX group. Besides, the RANKL/OPG ratio in OVX+BIBO3304 group was markedly decreased in comparison with that in OVX rats (Figure 4C, 4D). The results indicated that treatment with BIBO3304 inhibited the bone resorption, and promoted bone formation meanwhile in OVX rats.

### Characterization and identification of BMSCs

**Figure 5:**
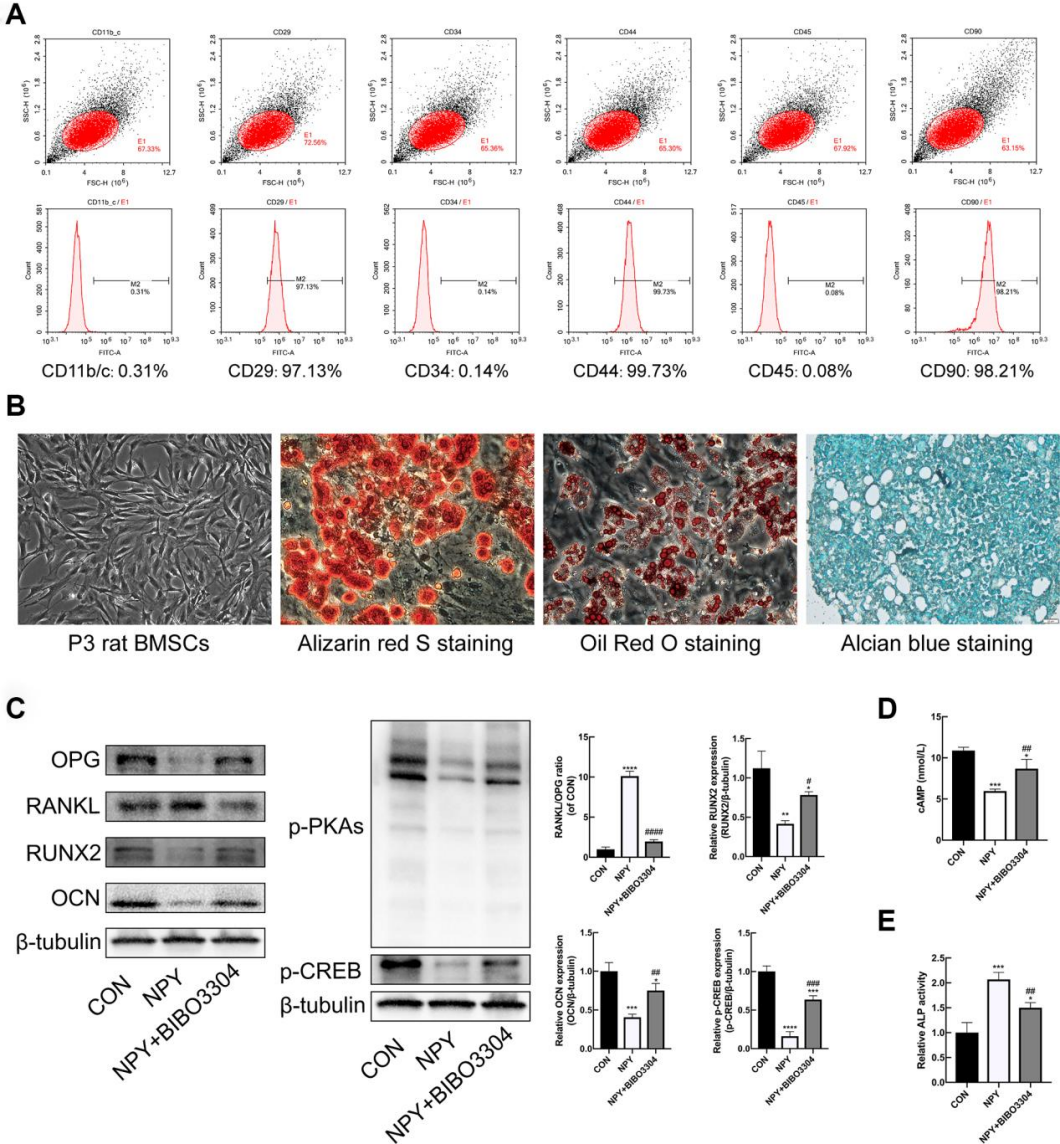
**Y1R antagonist treatment reversed NPY-mediated suppression of osteogenesis and cAMP/PKA/CREB pathway in BMSCs.** (**A**) Flow cytometric analysis of BMSC surface markers. (**B**) The multidifferentiation potential of BMSCs in vitro (Alizarin red S staining for osteogenic differentiation, Alcian blue staining for chondrogenic differentiation, and oil Red O staining for adipogenic differentiation). (**C**) Western blotting results of OPG, RANKL, RUNX2, OCN, p-PKAs and p-CREB expression in BMSCs after 3 days of incubation in osteogenic medium with NPY or NPY+BIBO3304. (**D**, **E**) The cAMP concentrations and ALP activity were measured on days 3 of incubation in osteogenic medium with NPY or NPY+BIBO3304. The data are expressed as the means ± SD. *P<0.05; **P<0.01; ***P<0.001; ****P<0.0001 vs. CON, and ^#^P<0.05; ^##^P<0.01; ^####^P<0.0001 vs. NPY by one-way ANOVA and Tukey’s post hoc test.

BMSCs at passage 3 were characterized by flow cytometry to detect the expression of the surface markers. As shown in Figure 5A, a high percentage of cells expressed the mesenchymal stem cell markers CD29, CD44, and CD90, and cells barely expressed the hematopoietic markers CD11b/C, CD45 and CD34. The Primary rat BMSCs displayed fibroblast-like morphologies under the microscope. And the BMSCs were successfully differentiated into osteogenic, adipogenic, and chondrogenic lineages in vitro under suitable differentiation conditions (Figure 5B). The results indicated that the cells we cultured were BMSCs with multilineage differentiation potential.

### Y1R antagonist treatment reversed NPY-mediated suppression of osteogenesis and cAMP/PKA/CREB pathway in BMSCs

With regard to the effects of NPY on BMSCs osteogenesis, BMSCs were cultured under osteogenic medium for 3d with and without the treatment of NPY or Y1R antagonist BIBO3304. The ALP activity was assessed among groups. And the protein expression levels of osteogenic marker RUNX2 and OCN, and the regulator of osteoclastogenesis RANKL/OPG were determined by western blotting. As shown in Figure 5C, 5E, the ALP activity was decreased, and the expressions of RUNX2 and OCN were significantly downregulated in NPY-treated BMSCs compared with the control group. Conversely, the RANKL/OPG ratio was higher in NPY-treated BMSCs. With the Y1R antagonist BIBO3304 treatment, the effects of NPY on BMSCs were reversed.

In addition, the expression of cAMP, p-PKAs and p-CREB were determined. The results indicated that NPY treatment clearly decreased the expression levels of cAMP, p-PKAs and p-CREB, and the decreases were partially attenuated by treatment with Y1R antagonist (Figure 5C, 5D). The results showed that NPY inhibited the BMSCs osteogenesis through activation of Y1R, which might be involved with the cAMP/PKA/CREB pathway.

### NPY inhibited osteogenesis and elevated RANKL/OPG ratio through Y1R via suppression of cAMP/PKA/CREB pathway in BMSCs

**Figure 6:**
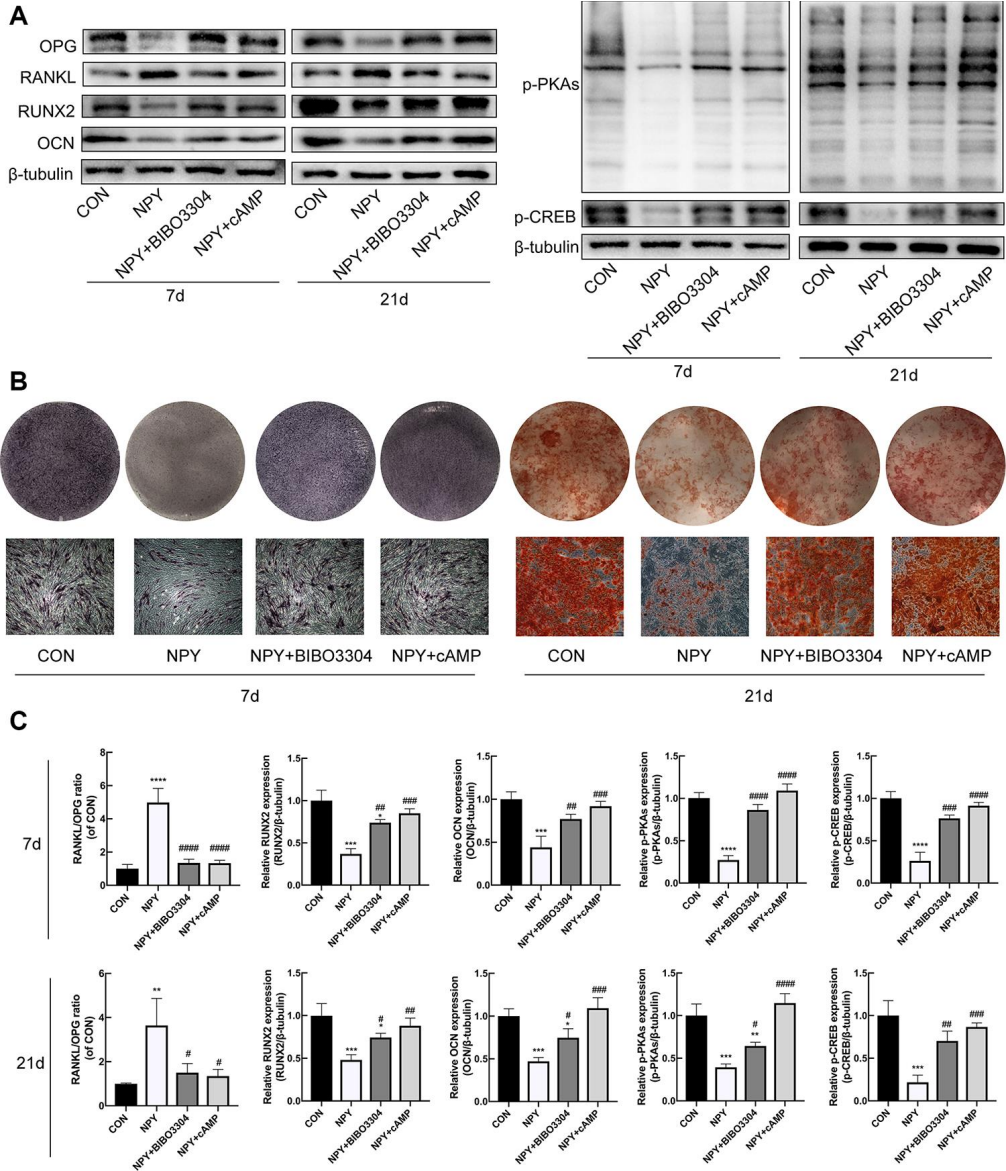
**NPY inhibited osteogenesis and elevated RANKL/OPG ratio through Y1R via suppression of cAMP/PKA/CREB pathway in BMSCs.** (**A**, **C**) Western blotting results of OPG, RANKL, RUNX2, OCN, p-PKAs and p-CREB expression in BMSCs after day 7 and 21 of incubation in osteogenic medium with NPY, NPY+BIBO3304 or NPY+cAMP. (**B**) ALP staining on day 7, and Alizarin red S staining of BMSCs on day 21 of incubation in osteogenic medium with NPY, NPY+BIBO3304 or NPY+cAMP. The data are expressed as the means ± SD. *P<0.05; **P<0.01; ***P<0.001; ****P<0.0001 vs. CON, and ^#^P<0.05; ^##^P<0.01; ^###^P<0.001; ^####^P<0.0001 vs. NPY by one-way ANOVA and Tukey’s post hoc test.

To investigate the relationship between the cAMP/PKA/CREB pathway and the Y1R in BMSCs, cells were treated with either the Y1R antagonist BIBO3304 or the PKA activator 8-Bromo-cAMP for 7d and 21d with the addition of NPY. Firstly, we evaluated the expression of RUNX2 and OCN, RANKL/OPG, and p-PKAs and p-CREB by western blotting. As shown in Figure 6A, 6C, NPY downregulated the expression levels of osteogenic marker RUNX2 and OCN, and p-PKAs and p-CREB at days 7 and 21, and increased the RANKL/OPG ratio. Both the BIBO3304 and 8-Bromo-cAMP reduced the effects of NPY. And then we evaluated the osteogenesis and mineralization of BMSCs through ALP and Alizarin Red S staining. Similarly, treatment with both the BIBO3304 and 8-Bromo-cAMP significantly attenuated the inhibitory effects of NPY on the osteogenesis of BMSCs at day 7, and mineralization at day 21 (Figure 6B).

## DISCUSSION

The present study demonstrated several novel findings through clinical samples, rat model and cellular experiments. First, OP patients showed more bone microdamage and upregulated expressions of NPY and Y1R in bone. Second, Y1R antagonist treatment improved bone microstructure and decrease microdamage in OVX rats. Third, Y1R antagonist treatment in vivo promoted bone formation and inhibit bone resorption in OVX rats. Finally, in vitro, NPY inhibited osteogenesis and elevated RANKL/OPG ratio through Y1R via suppression of cAMP/PKA/CREB pathway in BMSCs. These results suggest that NPY inhibits the cAMP/PKA/CREB pathway in BMSCs through Y1R, and Y1R antagonist plays an anti-osteoporotic effect and promoted microdamage repair in ovariectomized rats.

Consistent with our previous studies [7, 23], this study showed lower BV/TV and more bone microdamage in OP when compared with OA. BV/TV is one of the most important parameters of bone microstructure, which describing how many trabeculae there are in the bone. Our results showed that microcracks would be more numerous in lower BV/TV. For a given apparent stress, each bony element would experience a greater proportion of the apparent stress and consequently higher strain and likelihood of microcracking than a sample with higher BV/TV. Therefore, the osteoporotic bone is susceptible to fatigue loading due to insufficient structure. Except the bone microstructure, what we concerned is that there is significant difference in bone metabolism between OP and OA patients. Studies [24, 25] have demonstrated that OCN (a bone formation marker) was significantly lower and deoxypyridinoline (a bone resorption marker) was significantly higher in the OP group than those in the OA group for both bone and serum. And RANKL/OPG ratio in macerate of bone biopsies [26, 27] was higher in patients with osteoporosis in respect to that with osteoarthrosis. Giner et al. [28] revealed that osteoblasts from OP patients presented a higher RANKL/OPG ratio in response to 1,25-dihydroxyvitamin D than that from OA patients. All these studies suggested that OA group presented higher bone formation and lower bone resorption than OP group. Bone microdamage is repaired through bone remodeling, which is carried out by bone formation and bone resorption. To regulate the bone metabolism could both influence the microdamage repairing and the bone microstructure, which is implicated in resisting fatigue-induced microdamage. We previously [29] found that the NPY immunoreactivity in cancellous bone was significantly lower in the OA patients than that in the OP patients. Similarly, Liu et al. [30] demonstrated that the gene expression of NPY and Y1R in bone were higher in the OVX rats. It was suggested that the pathogenesis of PMO was associated with the regulation of NPY. The mechanism underlying the imbalance in NPY-Y1R signaling in PMO is still unclear. Peripherally NPY has been found in the sympathetic nervous system, which is co-stored and released with noradrenaline [31]. Studies demonstrated that sympathetic nerve discharge was increased in postmenopausal women, and estrogen administration could inhibit the sympathetic activity both in postmenopausal women and OVX rats [32, 33]. We supposed that the increased sympathetic activity in PMO may be involved in the imbalanced NPY-Y1R signaling. NPY exerts its function in bone homeostasis through Y1R and Y2R, which are the two most common receptors for NPY. Y1R could be detected in bone tissues, whereas Y2R was restricted to the brain [13]. In bone tissues, NPY could expressed by osteoblast lineage cells, BMSCs and peripheral nerve fibers [13, 15]. In the present study, we found that NPY and Y1R immunoreactivity in bone were stronger in the OP patients and OVX rats. In addition, the expression of NPY and Y1R in bone were negatively correlated with BV/TV in OP group. These results indicate that NPY-Y1R signaling pathway in the bone tissue might be a critical regulator of bone homeostasis. Germline Y1R knockout mice presented a higher bone mass phenotype and bone formation than those in wild-type mice [15]. It was suggested that NPY could suppress the bone formation through Y1R. In this study, we observed that the treatment of OVX rats with Y1R antagonist BIBO3304 alleviated the osteoporotic changes induced by ovariectomy, which were characterized by the improvement of bone mass and microstructure. This result was consistent with our previous study [34]. As expected, the OVX rats showed lower bone formation and higher bone resorption activity, which has been implicated in the bone loss induced by ovariectomy. And the treatment of OVX rats with Y1R antagonist BIBO3304 significantly lowered the bone resorption activity and elevated the bone formation. Our results revealed that blocking Y1R exerted an anti-osteoporotic effect on OVX rat, which might be a potential target for the treatment of PMO.

Microdamage occurs when bone tissues are under physiological loading, which can be repaired with a dynamic balance between the generation and repair of microdamage in healthy bone tissues [6]. The accumulation of microdamage has been implicated as an important factor which contributes to osteoporotic fracture [6]. In the present study, rats were all underwent the in vivo fatigue loading to induce the microdamage, and a greater amount of microdamage was found in OVX group. And Y1R antagonist treatment for OVX rats significantly decreased the number and length of microdamage. The associations between bone microstructure, bone remodeling and microdamage have been investigated in several studies [29, 35, 36]. The degraded bone microstructure in osteoporosis weakens the resistance to fatigue loading, and unbalanced bone remodeling impairs the microdamage repairing, which results in microdamage accumulation. Taken together, our results suggested that the Y1R antagonist treatment could reduce the accumulation of microdamage, which might decrease the risk of osteoporotic fracture.

To investigate the mechanisms underlying the regulation of bone metabolism by Y1R in bone, we evaluated the bone formation and bone resorption activity in bone marrow extracted from rats. Our data showed that the levels of osteogenic markers were significantly higher, and the osteoclastogenic markers were significantly lower in OVX+BIBO3304 group than those in OVX group. Given that the in vivo experiment showed encouraging results, we further investigated the mechanisms of Y1R on BMSCs in vitro. Previous studies [12, 14] have demonstrated that BMSCs expressed Y1R but not Y2R, suggesting that Y1R may mediate effects on bone via direct actions on BMSCs. BMSCs are multipotential stem cells that can be induced to differentiate into osteoblasts [37], and express the cytokines which play important roles in differentiation and recruitment of osteoclast precursor cells [38, 39]. It was suggested that NPY signaling via the Y1 receptor has been implicated in the regulation of BMSCs. Studies have demonstrated that treatment of BMSC with NPY markedly reduced the cell numbers [15] and osteogenic markers [13]. And BMSCs isolated from Y1R deletion mice showed significantly greater mineralization under osteogenic conditions than the BMSCs isolated from wild-type mice. Consistent with previous observations, we found that BMSCs cultured in the presence of NPY exhibited decreased expression of markers of osteoblast differentiation like OCN and RUNX2, and increased the RANKL/OPG ratio, which could be reversed by the treatment of Y1R antagonist. Both RANKL and OPG are expressed by BMSCs [39], and the RANKL/OPG system is regarded as a master regulator of the osteoclast regulatory pathway, which higher RANKL/OPG ratio could facilitate the differentiation of osteoclast precursor cells into mature osteoclasts [40]. Our results suggested that NPY-Y1R signaling suppressed the osteoblast differentiation of BMSCs, and facilitate the osteoclastogenesis through elevating the RANKL/OPG ratio.

The Y1R belongs to G-protein-coupled receptor and have been shown to decrease the isoprenaline-induced cAMP levels of mouse BMSCs [41], and NPY treatment strongly suppressed cAMP/PKA pathway and phosphorylation of CREB through Y1R in mouse mesenchymal stem cells. The anabolic function of cAMP/PKA/CREB signaling pathway in bone has been characterized in numerous studies [16–20, 42]. Zhang et al. [42] revealed that parathyroid hormone activated cAMP/CREB in osteoblasts, promoting osteoblastic differentiation. And the activation of cAMP/PKA/CREB signaling pathway in human BMSCs contributed to the osteogenic effects with the upregulated level of Runx2 [16]. Furthermore, the activation of cAMP/PKA pathway in mesenchymal stem cells and osteoblasts has been found to resulted in the decreased RANKL/OPG ratio, which inhibited the osteoclastogenesis [19]. We therefore hypothesized that the mechanism underlying NPY-Y1R regulation of BMSCs differentiation lies in the inhibition of cAMP/PKA/CREB pathway. To test this hypothesis, first we cultured BMSCs in the presence of NPY with or without Y1R antagonist. Consistent with previous observations, we found that NPY treatment decreased the cAMP levels in BMSCs, which could be reversed by Y1R antagonist BIBO3304. Besides, Y1R antagonist blocked the effects of NPY on inhibiting the phosphorylation of PKA and CREB. Next, we further cultured BMSCs with or without 8-Bromo-cAMP, an activator of PKA, in the presence of NPY or Y1R antagonist BIBO3304. We found that 8-Bromo-cAMP could significantly reverse the inhibitory effect of NPY on cAMP levels and phosphorylation of PKA and CREB in BMSCs which mediated through Y1R. Our results suggested that NPY inhibited the cAMP/PKA/CREB pathway through Y1R in BMSCs. Previous studies have demonstrated that the activation of cAMP/PKA/CREB signaling pathway in human BMSCs [16] and osteoblasts [17, 18] contributed to the osteogenic effects with the upregulated level of Runx2. And studies found that the activation of cAMP/PKA pathway in mesenchymal stem cells [19] and osteoblasts [20] resulted in the decreased ratio of RANKL/OPG. Our results showed that NPY treatment suppressed the osteoblast differentiation of BMSCs, and facilitate the osteoclastogenesis through elevating the RANKL/OPG ratio, which could be reversed by either the Y1R antagonist or the PKA pathway agonist treatment. Taken together, these observations suggest NPY-Y1R inhibit osteoblast differentiation of BMSCs and elevate the RANKL/OPG ratio via cAMP/PKA/CREB pathway.

Currently, the most used therapeutic agents for osteoporosis are anti-resorptive drugs such as bisphosphonates and denosumab. These agents inhibit bone resorptive function of osteoclasts, while do not enhance bone formation and foster the development of new bone. Long-term suppression of bone remodeling may result in the accumulation of bone microdamage, which compromises the mechanical properties of bone [43, 44]. Our results showed that Y1R antagonist treatment could enhance bone formation and inhibit bone resorption, which ultimately improved osteoporosis and reduce the accumulation of microdamage. It was suggested that regulation of NPY-Y1R in bone metabolism might be a potential therapy strategy for the prevention of osteoporosis and osteoporotic fracture. It's worth noting that there might be several potential long-term side effects of Y1R antagonist treatment as well. On the one side, NPY is one of the most potent regulators of food intake and energy homeostasis [45–47], and long-term treatment of Y1R antagonist may inhibit the feeding behavior. On the other side, long-term improper dose of Y1R antagonist treatment may cause the oversuppression of bone resorption or overactivity of bone formation, which may result in the disorder of bone remodeling and the accumulation of microdamage. Therefore, further research on the local regulation of NPY-Y1R in bone and the appropriate long-term dose of Y1R antagonist treatment are needed.

This study still has some limitations that should be acknowledged. First, although we performed the sample size estimation on the basis of the previously published data, the sample size was small. In this study we included and excluded the patients in strict accordance with criteria. Second, the clinical sample experiment in this study was an exploratory study. For it is difficult to obtain the healthy people’s bone as normal control group, therefore we used patients with osteoarthritis of the hip as the control group.

In conclusion, our findings have demonstrated that NPY-Y1R can inhibit osteoblast differentiation of BMSCs and elevate the RANKL/OPG ratio via cAMP/PKA/CREB pathway. Y1R antagonist treatment in vivo for OVX rats could improve the bone microstructure and decrease bone microdamage. This study highlights the regulation of NPY-Y1R in bone metabolism as a potential therapy strategy for the prevention of PMO and osteoporotic fracture.

## MATERIALS AND METHODS

### Clinical specimens

The subchondral cancellous bone specimens were obtained from 10 patients who underwent total hip arthroplasty for primary osteoarthritis OA (mean age 69, range 63–78 years) and 10 patients who underwent total hip arthroplasty for hip osteoporotic fracture (mean age 68.3, range 61–77 years). The sample size estimation was calculated on the basis of the previously published data [23] comparing BV/TV and trabecular thickness (Tb.Th) in OA compared with OP. Setting the power at 0.90, the significance level at 0.05 with two-tailed testing, the maximum calculated sample size was 9. Thus we set a sample size of 10 patients in each group. To avoid the confounding effect of age and sex, postmenopausal women with a history of menopause for more than 5 years were included. The inclusion and exclusion criteria in this study followed as published previously [7, 23, 29]. Briefly, OA patients with grade III-IV disease according to the Outerbridge classification [48] and OP patients with femoral neck fracture were included. Patients with other osteoarticular diseases like congenital or acquired dysplasia and rheumatoid arthritis were excluded. And patients with diseases and therapy that influence bone metabolism were excluded as well. Informed consent was obtained from all individual participants included in the study. This study was approved by the Institutional Review Board of Shanghai Renji Hospital.

The specimen preparations were carried out as described previously [7, 23, 29]. Briefly, the femoral head was obtained from each included patient underwent total hip arthroplasty, and two bigger samples (15×15mm in cross-section and 10mm long) of subchondral cancellous bone were obtained from the load-bearing area (Figure 1A) for micro-computed tomography (micro-CT) imaging and microdamage evaluation. And one smaller sample (5×5mm in cross-section and 10mm long) was obtained for immunohistochemical analysis.

### Animal groups

The animal experiments were ethically approved by the Institutional Animal Care and Use Committee of Shanghai Jiaotong University and was conducted by the Guide for the Care and Use of Laboratory Animals of the National Institutes of Health. Eighteen 3-month-old female virgin SD rats were randomly divided into 3 groups (n=6 per group): the SHAM group was a control group containing sham-operation rats; the rats in OVX group were ovariectomized and treated with 0.9% saline; the OVX+BIBO3304 group was a treatment group in which OVX rats were treated with Y1R antagonist BIBO3304 (Tocris Bioscience, Bristol, UK) (1 mg/kg/day, i.p.). For OVX rats, bilateral ovaries were excised, and for SHAM rats, adipose tissue of the equal weight as the ovaries was removed. After 8 weeks following surgery, drugs were administered to rats daily for 4 weeks.

### In vivo fatigue loading

In vivo fatigue loading was performed to induce bone microdamage in all animals. In 12 weeks after operation, the left tibias of all rats were subjected to fatigue loading as previously described [49]. In brief, left knees and ankle joints were placed in concaved cups of the mechanical testing machine (Instron 8874; Instron Limited). Dynamic compressive loads (peak loads of 70 N, 4 Hz, sinusoidal wave) were applied axially to the tibias. The load was applied 1,4400 times per day for 2 d. All rats were euthanized 3d after fatigue loading. The left tibias were fixed in 70% ethanol for microdamage evaluation. The right tibias were collected for micro-CT imaging. The right femurs were fixed in 4% paraformaldehyde for immunohistochemical analysis. Besides, the bone marrow of left femurs was collected. After erythrolysis by Red Blood Cell Lysis Buffer (Sigma Aldrich), the total protein was extracted using RIPA lysis buffer (Beyotime, Shanghai, China). The protein of bone marrow was frozen at −80°C for western blot.

### Bone micro-computed tomography (micro-CT) imaging

The clinical bone specimens and right tibia of rats were examined using a micro-CT system (SkyScan-1176, Bruker microCT, Belgium) at a voxel size of 18 μm. After scanning, a region of interest (ROI) centered over the specimen was selected for analysis. The three-dimensional (3-D) reconstruction of trabecular bone was performed according to the ROI. The quantitative analysis was carried out and the following parameters were calculated by the software provided with the instrument: Bone mineral density (BMD, g/cm^3^), bone volume fraction (BV/TV; %), trabecular number (Tb.N; mm^−1^), trabecular thickness (Tb.Th; μm), trabecular separation (Tb.Sp; μm), connectivity density (Conn.D; mm^−3^) and structure Model Index (SMI).

### Microdamage evaluation

The microdamage of clinical bone specimens and left tibia of rats was evaluated according to the method reported previously [50]. Briefly, the specimens were fixed in 70% ethanol for more than 48h and bulk stained in 1% basic fuchsin in a graded series of ethanol under vacuum. And then the specimens were embedded in polymethylmethacrylate. The middle segment of tibia specimens was longitudinally sectioned into 60 μm-thick slices. Slides were observed using green incident light (λ=594 nm) and microdamage identified by red fluorescence. And under ultraviolet incident light (λ=365 nm), microdamage identified by purple fluorescence. The crack length (Cr.Le; μm) was initially examined, and then crack numerical density (Cr.Dn; #/mm^2^) and crack surface density (Cr.S.Dn; μm/mm^2^) were calculated to evaluate the microdamage of the specimens.

### Immunohistochemistry

The clinical bone specimens and right distal femurs of rats were embedded with paraffin after decalcification and were sectioned into 5-mm thickness. The immunohistochemistry was assessed according to the method reported previously [29]. Briefly, the sections were incubated with the primary antibody at 4°C overnight, and then with a biotinylated secondary antibody for 15 min at 37°C. The primary antibodies for the clinical bone specimens included rabbit anti-NPY (1:200, Abcam, Cambridge, USA), rabbit anti-NPY1R (1:200, Abcam, Cambridge, USA) or rabbit anti-NPY2R (1:200, Abcam, Cambridge, USA) antibody. And the sections of rat femurs were stained with rabbit anti-Runx2 (1:200, Affinity Biosciences, Inc. USA) or rabbit anti-matrix metalloproteinase-9 (MMP9, 1:200, Affinity Biosciences, Inc. USA). Peroxidase reaction was visualized using a solution of diaminobenzidine. The images were obtained with a Spot Advanced digital-imaging system (Diagnostic Instruments, Inc., Sterling Heights, MI, USA), and the mean optical density (MOD) was measured using the software package Image Pro Plus (version 5.0.1, Media Cybernetics, Silver Spring, MD, USA).

### Histological examination

The sections of distal rat femurs were stained with hematoxylin and eosin (HE, Beyotime Biotech, Shanghai, China), tartrate-resistant acid phosphatase (TRAP, Sigma-Aldrich, St. Louis, MO, USA) and Alizarin Red (Sigma-Aldrich) staining. The staining was performed according to manufacturer's protocol and finally observed using a digital microscope (Olympus, Japan).

### Isolation, culture, identification and differentiation of rat BMSCs

Primary BMSCs were harvested from 4-week-old SD rats as described previously [51]. Briefly, bone marrow was flushed out from the femurs with DMEM/F12 (Gibco, Grand Island, NY, USA) supplemented with 10% fetal bovine serum (FBS; Gibco, Grand Island, NY, USA), 100 U/ml penicillin, and 100 μg/ml streptomycin (Gibco, Grand Island, NY, USA). After being centrifuged at 800 rpm for 5 min, the cells were cultured in 25 cm^2^ culture flasks (Corning, NY, USA) at 37°C with 5% CO2. The medium was changed every 2 days, and the non-adherent cells were removed. When the confluence reached approximately 80%, the adherent cells were harvested by incubation with 0.25% trypsin-EDTA (Gibco, Grand Island, NY, USA) and were passaged. BMSCs at passage 3 were identified by flow cytometry to detect the expression of the surface markers CD29, CD90, CD44, CD34, CD45 and CD11b. To confirm the multilineage differentiation potential, the BMSCs were cultured under adipogenic, chondrogenic, and osteogenic differentiation conditions, and stained with oil Red O (Sigma-Aldrich, USA), Alcian blue (Sigma-Aldrich, USA), and Alizarin Red (Sigma-Aldrich, USA), respectively. The multilineage differentiation examination were performed described previously [51]. BMSCs at passage 3-5 were used for experiments.

### Cell treatment and groups

BMSCs were seeded in 6-well plates (Corning, NY, USA) at 1 × 10^5^ cells/well. we divided the cells into 4 groups to investigate the role of NPY in the cAMP/PKA signaling pathway and the Y1R relationship. The CON group was treated a volume of phosphate-buffered saline (PBS; Gibco, Grand Island, NY, USA) equal to that used for treatments; The NPY group was treated with 10^-10^ M NPY (Abcam, Cambridge, USA); The NPY+BIBO3304 group was supplemented with 10^-10^ M NPY and 10^-6^ M Y1R antagonist (BIBO3304, Tocris Bioscience, UK). The NPY+ cAMP group was treated with a mixture of NPY and 10^-4^ M 8-bromoadenosine-3',5'-cyclic monophosphate (8-Bromo-cAMP, Selleck Chemicals, USA). 8-Bromo-cAMP is a small molecule cAMP analogue, which is an activator of PKA. For osteogenic induction, the medium was changed to the osteogenic medium containing with 10^-7^ M dexamethasone (Sigma-Aldrich, USA), 10^-2^ M sodium β-glycerophosphate (Sigma-Aldrich, USA) and 50 μg/mL L-ascorbic acids (Sigma-Aldrich, USA).

### ALP activity assay

BMSCs were cultured with osteogenic medium for 3d. ALP activity was quantified in cell lysates using an ALP assay kit (Beyotime Biotech, Shanghai, China) according to the manufacturer’s instructions.

### Enzyme linked immunosorbent assay (ELISA)

The cAMP concentrations in the media were measured. BMSCs were cultured with osteogenic medium for 3d. The culture media were collected and centrifuged at 1000rpm for 10min, and the supernatants were stored at -80°C until use. The cAMP concentration was detected using Rat cAMP ELISA kit (Jianglai biotech, Shanghai, China), according to the manufacturer’s instructions. The limit of detection was 0.5-20 nmol/L, and the coefficient of variation (CV) was less than 9%. The absorbance was read at 450 nm using a microplate reader. The concentrations were calculated according to the optical density (OD) and standard curve.

### Alizarin Red S and ALP staining

After osteogenic induction for 7d, cells in 6-well plates were stained by Leukocyte Alkaline Phosphatase Kits (Sigma-Aldrich, USA) for ALP staining. And Alizarin Red S staining was performed to detect the mineralized nodule formation after BMSCs were incubated with osteogenic medium for 21d. All the staining was performed according to the manufacturer’s instructions.

### Western blot

BMSCs were cultured with osteogenic medium for 3d, 7d and 21d. Total protein of BMSCs or rat bone marrow was extracted using RIPA lysis buffer (Beyotime, Shanghai, China). The protein concentration was measured using the bicinchoninic acid protein assay kit (Thermo Scientific, Rockford, IL, USA). Proteins were separated using SDS-PAGE gel and then transferred to 0.22 μm polyvinylidine diluoride membrane (Millipore, USA). Membranes were incubated at 4°C overnight with a 1:1000 dilution primary antibodies for RANKL (1:1000; Santa Cruz, Biotechnology, Inc. USA), OPG (1:1000; Affinity Biosciences, Inc. USA), Osteocalcin (OCN, 1:1000; Affinity Biosciences, Inc. USA), RUNX2 (1:1000; Affinity Biosciences, Inc. USA), MMP9 (1:1000; Affinity Biosciences, Inc. USA), Cathepsin K (Cath-K, 1:1000; Affinity Biosciences, Inc. USA), GAPDH (1:1000; Proteintech, Chicago, IL, United States) or β-tubulin (1:1000; Proteintech, Chicago, IL, United States), and then incubated for 1 h at room temperature by secondary antibody. The immunoblots were visualized using enhanced chemiluminescence (Thermo Scientific) and images were obtained using ChemiDoc XRS (Bio-Rad, USA).

### Statistical analysis

Data were analyzed using GraphPad Prism software (GraphPad Prism 8.00, San Diego, USA). All data were presented as the means ± standard deviation (SD). The difference between two groups was evaluated by the Student’s t-test, and One-way analysis of variance was used for the statistical comparisons between multiple groups. Pearson correlation analysis was performed to evaluate the correlation between variables. The significance level was set at P < 0.05.
